# Thermal Niche Differentiation Shapes the Hibernating Bat Assemblages in Bulgarian Caves Across an Elevational Gradient

**DOI:** 10.3390/biology15060484

**Published:** 2026-03-19

**Authors:** Heliana Dundarova, Ilya Acosta-Pankov, Elena Nedyalkova, Andrea Lubenova, Maksim Kolev, Krasimir Kirov, Krasimir Lakovski, Olya Genova, Valeri Parvanov, Plamenka Iskrenova, Vladimir Trifonov, Tsenka Chassovnikarova

**Affiliations:** 1 Institute of Biodiversity and Ecosystem Research, Bulgarian Academy of Sciences, 1000 Sofia, Bulgaria; 2 National Museum of Natural History, Bulgarian Academy of Sciences, 1000 Sofia, Bulgaria; 3 Faculty of Biology, Sofia University “St. Kliment Ohridski”, 1000 Sofia, Bulgaria; 4 Department of Natural History, Rousse Regional Museum of History, 7000 Ruse, Bulgaria; 5 Vrachanski Balkan Nature Park Directorate, 3000 Vratsa, Bulgaria; 6 Regional Inspectorate of Environment and Water (RIEW)—Montana, 3400 Montana, Bulgaria; 7 Regional Inspectorate of Environment and Water (RIEW)—Haskovo, 6300 Haskovo, Bulgaria; 8 Department of Ecology and Environmental Conservation, Faculty of Biology, University of Plovdiv “Paisii Hilendarski”, 4000 Plovdiv, Bulgaria

**Keywords:** elevation gradient, bat assemblages, thermal niche, species turnover, climate change, environmental filtering, indicator species

## Abstract

Elevation is a strong proxy for the thermal environment because it causes a predictable drop in temperature and food availability. This restricts cave-dwelling bats to species with specific metabolic traits, such as torpor or migration to avoid cold stress. In this context, we aimed to reveal how thermal niche differentiation structures 25 cave-dwelling bat assemblages along elevation gradients in two of the largest Bulgarian mountains—Stara Planina and Rhodopi. Multivariate PERMANOVA showed significant differences in bat assemblages among elevation groups (F = 1.616, *p* = 0.046), with altitude and temperature explaining 32.4% of the variance (*p* = 0.001). A high degree of species turnover (91.12% dissimilarity), driven by temperature niches, was observed: mesophilic *Rhinolophus* species dominated warm, low-elevation caves, while cold-adapted *Myotis* species were more common at high elevations. SIMPER analysis identified *R. euryale* as an indicator in low-elevation caves (*p* = 0.012) and the *M. myotis/blythii* complex at high elevations (*p* = 0.003). Alpha diversity showed no variation across elevation groups (*p* = 0.293), indicating that species turnover occurs without overall changes to local diversity. Mid-elevation assemblages lacked specific indicator species and resembled high-elevation communities, forming an ecotone. Thermal niche partitioning, as a physiological filter, shapes cave-dwelling bat assemblages and affects climate change range-shift predictions.

## Simple Summary

Bats in temperate areas enter hibernation, a survival tactic that helps them to reduce metabolic expenses during winter, when food is scarce. In this study, we investigate hibernating bat assemblages from different caves along the elevation gradient in two of Bulgaria’s largest mountain ranges: the Stara Planina Mountains and the Rhodopi Mountains. We aimed to reveal how elevation-associated temperature influences the species composition of the cave assemblages. Our results show that each bat species has distinct temperature preferences that determine where it hibernates. Species that prefer warmer conditions inhabited low-elevation caves, whereas those adapted to colder temperatures occupied high-elevation caves. Interestingly, the total number of species remained relatively stable across elevations, suggesting that species replace one another rather than diversity declining simply with elevation. Caves at mid-elevations hosted mixed communities without any single characteristic species, indicating that mid-elevations function as transition zones. Overall, our findings demonstrate that temperature preferences shape which bat species hibernate together. This has important implications: as climate warming continues, temperature-specialist bat species may be forced to shift their elevational ranges to find suitable hibernation sites.

## 1. Introduction

In temperate regions, caves serve as essential refuges for bat hibernation, providing more stable thermal environments than surface habitats [1,2,3], thereby reducing energetic costs during winter [4,5]. The spatial heterogeneity of caves, including tunnel systems, chambers, entrances, and fissures, creates diverse microclimatic zones. These zones support species with different thermoregulation strategies [1,6]. Varying temperatures and conditions in different cave areas have allowed each bat species to select the most suitable spot [6]. Some prefer warm, stable conditions deep inside caves, while others choose cooler zones near entrances or areas with temperature fluctuations [7,8]. This enables multiple species to share the same hibernation area without overlapping thermal requirements, reducing thermal competition among them. By occupying distinct microhabitats, species coexist without interference, thereby increasing the cave’s capacity to support diverse bat communities [1,6,9].

To survive winter, temperate bats rely on extended torpor, a complex physiological adaptation shaped by evolutionary history [10]. This strategy conserves energy when insect prey is unavailable [11]. Energy savings depend directly on the duration and depth of torpor [12]. During torpor, metabolic rate decreases substantially, and body temperature approaches ambient conditions, often matching the temperature of the cave walls. While low temperatures maximize energetic efficiency, maintaining body temperature above freezing is critical to prevent tissue damage [13,14]. Consequently, the roost microclimate directly influences energy budgets [14]. Vital functions, such as fat metabolism and arousal frequency, are tightly coupled to ambient temperature [6,15]. Although optimal hibernation temperatures typically range from 2–12 °C, this varies with species’ body size and local environmental conditions [16]. Consequently, hibernation strategies are species-specific, varying in thermal optima, cold tolerance, and torpor depth [7].

Bats with varying thermal physiologies inhabit diverse microhabitats. This leads to thermal niche differentiation that may influence how multiple species cohabit at shared hibernation sites. Temperature generally drops by about 0.6 °C per 100 m of elevation [17], creating a thermal gradient over relatively short distances. This gradient affects both the external climate near hibernation sites and the microclimates within subterranean roost sites [18]. Warmer hibernation sites are at lower elevations; cooler subterranean roosts are at higher elevations, where external temperatures are lower, although internal thermal variation persists [19]. Bat assemblages change along elevation gradients, forming a vertical zonation in which warm-preferring species inhabit lower elevations and cold-tolerant species occupy higher elevations [19]. This turnover reflects environmental filtering driven by physiological tolerances and thermal niche differentiation. Importantly, this pattern persists despite internal thermal heterogeneity within individual caves. Beyond temperature, however, humidity remains a critical factor; bats must find environments sufficiently damp to prevent dehydration from water loss through their wings and respiratory systems [15,20]. Other environmental factors can also influence hibernation site selection, including airflow, thermal stability, and chamber morphology [1]. Temperature is the most widely documented and physiologically critical variable in hibernating bats [6]. We therefore focus on temperature as the primary environmental axis while acknowledging that other unmeasured factors may also contribute to assemblage structure.

Several studies have demonstrated that elevation acts as a thermal filter, grouping species into zones aligned with their physiological needs. In the Southern Poland Carpathians, bat distribution exhibits a non-linear pattern, with sharp changes in species composition at specific altitudes [19], while in Romania, cave temperature and location are also key factors determining species presence [7]. Ultimately, hibernating bat assemblages shift predictably with altitude. The clear distinction in thermal niches becomes evident when analyzing the bats’ actual body temperatures in relation to their roosting sites’ microclimates: *Murina hilgendorfi* hibernates at a cold-hardy 2.6 °C, while *Myotis formosus* to a much warmer 13.6 °C [21]. Predictably, species distribution across the landscape is based on the best match between the subterranean ‘thermal zone’ and their physiological limits.

The species-specific torpor physiology determines the thermal niche partitioning. Beyond simple temperature preferences, bats differ in their metabolic and evaporative water-loss rates. The latter dictates how often they must arouse to rehydrate [22,23]. Interestingly, these physiological traits are not fixed. Populations of the same species frequently show distinct adaptations influenced by their geographic location, with southern populations generally having higher metabolic rates than northern ones [24]. This suggests that a bat’s thermal niche is not a fixed trait, but a flexible evolutionary response to local environmental pressures like elevation.

Despite extensive research on bat hibernation ecology over several decades, significant knowledge gaps remain regarding the relative importance of different cave characteristics, species-specific preferences, and how these relationships vary across geographic regions and climatic zones [3,25]. Most previous studies have focused mainly on how elevation gradients influence species composition in forest-dwelling bat communities [7]. However, factors such as habitat degradation, loss, disease transmission, and climate change are driving declines in bat abundance and species diversity [26]. Thus, investigating how elevation affects the thermal niches of hibernating bats is crucial for their conservation. Climate change-induced shifts in the temperature of bat hibernation sites could reduce the availability of suitable roosts, forcing bats to use less optimal sites or abandon traditional hibernation locations [27]. Such changes can alter bat communities, with some species disappearing from certain areas and others expanding into new regions [28]. In this context, it is becoming more urgent to understand how environmental gradients in the mountains influence the distribution patterns of cave-dwelling bats [3,15].

Bulgaria hosts some of Europe’s largest hibernation colonies [29], establishing it as a biodiversity hotspot [30]. At least 10 cave-dwelling species have been documented, including multiple *Myotis*, *Rhinolophus*, and *Miniopterus* species [31,32,33,34]. Despite this taxonomic richness, significant knowledge gaps remain. Studies have employed diverse methods, including passive acoustics, visual counts, and fecal analysis. These efforts are site-specific and temporally limited [33,35]. No comprehensive assessment exists of how environmental gradients structure cave-dwelling bat assemblages at regional scales in Bulgaria. Specifically, the role of elevation-associated temperature in determining species composition and thermal niche partitioning remains unexplored. In this context, this study aimed to examine how thermal niche differentiation structures hibernating bat assemblages along an elevation gradient, identify the thermal requirements of different species, and predict how assemblages may respond to future changes in temperature. We examined cave-dwelling bat assemblages across an elevation gradient (100–1334 m) in Bulgaria to test whether thermal gradients structure assemblage composition through environmental filtering. We predicted that bat assemblages would differ significantly across elevation zones, with warm-adapted species dominating low elevations and cold-adapted species dominating high elevations. These patterns would be primarily driven by elevation-related temperature.

## 2. Materials and Methods

### 2.1. Material

#### 2.1.1. Study Region

**Figure 1:**
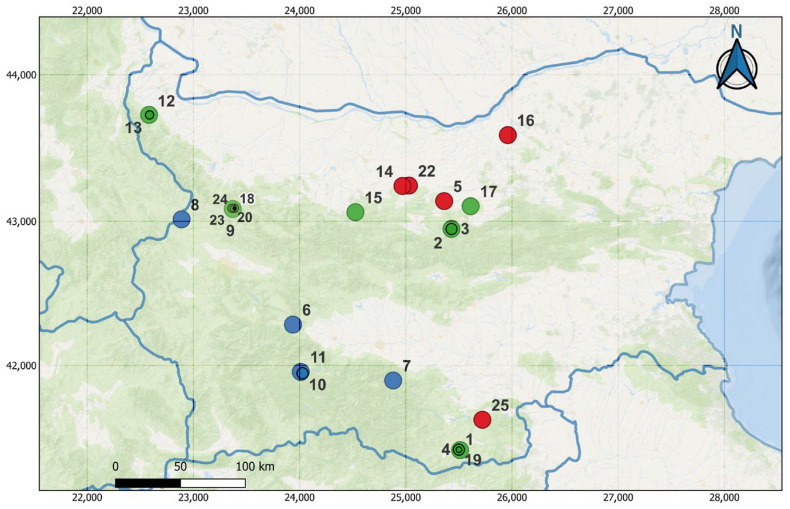
Topographic locations of the study subterranean sites in Bulgaria. Numbers (1–25) correspond to the Cave IDs of the investigated caves listed in Table 1. Each cave is marked with a colored point indicating its elevation: red for low elevation (100–300 m a.s.l.), green for medium elevation (330–600 m a.s.l.), and blue for high elevation (>680 m a.s.l.).

The study comprises the low and mid altitudes of two large mountain ranges: the Stara Planina Mountains and the Rhodope Mountains (Figure 1). The area is characterized by Mesozoic limestone formations with extensive karst development, supporting diverse cave systems across elevation zones [36]. The Stara Planina Mountain has a varied climate and distinct vertical plant belts, ranging from temperate in the lower foothills to cold at higher elevations. They act as a climatic barrier with high humidity and snow lasting up to four months, shaping ecosystems. Average temperatures range from 10 °C in the foothills to 0.7 °C on high ridges [37]. Vegetation shows “climatic compression,” with ecological zones within a narrow elevation range. The lowest zone includes xerothermic oak forests up to 1100 m, and the Beech Belt (1100–1500 m), dominated by *Fagus sylvatica*, forms dense forests [37].

**Table 1:** Geographical and Structural Characteristics of the Studied Hibernacula in Bulgaria.

Subterranean Sites	Cave ID	Latitude (N)	Longitude (E)	Altitude (m)	Roost Length (m)	Species Richness
Aina-Ini Cave	1	41.4	25.51	389	21	2
Andaka Cave	2	42.94	25.43	323	5000	7
Bacho Kiro Cave	3	42.94	25.43	335	3500	2
Cave next to Samara Cave	4	41.4	25.49	382	50	1
Emenska Cave	5	43.13	25.36	264	264	6
Golak mine	6	42.28	23.93	688	688	4
Ivanova Voda Cave	7	41.89	24.88	1336	657	2
Kalotinska Temna Dupka Cave	8	43.01	22.88	683	674	4
Kozarskata Cave	9	43.08	23.36	493	709	1
Lednikat Cave	10	41.94	24.02	1218	1390	4
Lepenitsa Cave	11	41.95	24.01	1073	600	5
Magura Cave	12	43.72	22.58	384	3645	4
Ringova Pesht Cave	13	43.72	22.58	365	200	2
Mandrata Cave	14	43.24	24.96	220	530	3
Mikrenska Cave	15	43.06	24.52	422	1921	1
Orlova Chuka Cave	16	43.59	25.96	130	13,437	6
Ovcharskata Cave	17	43.1	25.61	406	12	4
Razhishka Cave	18	43.09	23.38	503	316	4
Samara Cave	19	41.89	24.88	389	338	8
Svinskata Dupka Cave	20	43.08	23.37	472	383	3
Temnata Dupka Cave	21	43.08	23.38	435	9000	2
Urushka-Maara Cave	22	43.24	25.02	230	1600	2
Vrazhi Dupki 14 Cave	23	43.08	23.36	511	215	2
Vrazhi Dupki 9 Cave	24	43.08	23.36	488	43	2
Zandana Cave	25	41.61	25.72	177	76	3

The Rhodope Mountains in southern Bulgaria have a diverse climate, blending Mediterranean influences in the south and east with mountainous/continental conditions in the west and north. Temperatures vary with altitude and region: the Eastern Rhodopes are warmer, averaging 12–13 °C, while the Western Rhodopes have a montane climate, with temperatures ranging from 5 °C to 10 °C. Winter temperatures often drop to −15 °C [38]. Vegetation is shaped by the Mediterranean climate, creating a “climatic compression” of plant belts: xerothermic communities of *Quercus frainetto* and *Quercus cerris* at lower altitudes and southern slopes; valleys host mixed forests of *Quercus dalechampii*, *Fagus sylvatica*, and *Carpinus betulus*; middle altitudes have beech forests, often mixed with *Acer pseudoplatanus*, *Populus tremula*, and *Abies alba* [39].

#### 2.1.2. Subterranean Sites

The study was conducted at 24 caves and 1 mine in Bulgaria between 2020 and 2025, in accordance with approved European practices for bat research under licenses no. 760/15.11.2018, 828/19/03/2020, 912/02.11.2021, 960/06.12.2022, and 982/21.07.2023 from the Bulgarian Ministry of Environment and Water. All activities involving the capture and handling of animals were performed following ethical guidelines established by the Animal Ethics Committee of the Institute of Biodiversity and Ecosystem Research, Bulgarian Academy of Science, and were fully compliant with the European Parliament and Council directives regarding the protection of animals used for scientific purposes.

All surveyed caves are formed in Mesozoic limestone karst formations, which dominate both the Stara Planina and Rhodopi mountain ranges (Figure 1). The uniformity in lithology across sites eliminates rock type as a confounding variable in cave thermal environments. We selected caves based on known bat hibernation sites from previous surveys, winter accessibility, and representation across the elevation gradient. We monitored 15 caves in the Stara Planina Mountain (Ringova Pesht Cave, Kalotinska Temna Dupka, Kozarskata, Vrazhi Dupki 9, Vrazhi Dupki 14, Svinskata Dupka, Temnata Dupka, Razhishka, Mikrenska, Ovcharskata, Emenska, Andaka, Bacho Kiro, Urushka-Maara, Mandrata Caves) within the Natura 2000 protected zone designated under the EU Habitats Directive [40], and 1 cave (Magura) was designated a natural landmark. In the Rhodope Mountains, we monitored 7 caves: Ivanova Voda, Lednikat, Lepenitsa, Aina-Ini, Samara, the cave next to Samara, and Zandana Caves. In addition, we investigated the Golak mine in the Sredna Gora mountain and the Orlova Chuka Cave on the Danube Plateau (Figure 1). The geographical and structural characteristics of the hibernacula studied are shown in Table 1. These subterranean environments correspond to the natural habitat type 8310 (Caves not open to the public), listed in Annex I of the Council Directive 92/43/EEC [40] and in Annex I of the Bulgarian Biological Diversity Act [41], and are recognized as conservation priorities as critical reservoirs of biodiversity.

Caves were grouped into three elevation categories based on the distribution of cave sites along the elevation gradient (100–1334 m a.s.l.), the need for adequate replication in multivariate analyses, and observed thermal zonation in the study area, which affects the cave temperature. The resulting groups were: Low elevation (100–300 m a.s.l., *n*= 5 caves, mean = 204.2 ± 51.8 m), Mid elevation (330–600 m a.s.l., *n* = 15 caves, mean = 419.9 ± 61.3 m), and High elevation (>680 m a.s.l., *n* = 5 caves, mean = 999. 6 ± 301. 5 m). These elevation categories corresponded to significantly different thermal environments, with mean cave temperatures of 10.55 ± 1.38 °C (Low), 7.90 ± 1.62 °C (Mid), and 3.73 ± 1.59 °C (High) (ANOVA: F_2, 22_ = 11.59, *p* < 0.001). Cave characteristics (entrance size, passage length, chamber volume) were documented but not used as selection criteria to avoid confounding elevation with structural complexity.

### 2.2. Methods

#### 2.2.1. Hibernacula Survey and Counting Monitoring

We conducted single-visit surveys at each site during peak hibernation (January 2020–February 2025) to minimize disturbance while maximizing detection. The single-visit protocol followed best practices for hibernation surveys to prevent arousal-induced mortality from repeated disturbance. Survey timing was standardized (10:00–16:00) to control the temperature variation. All accessible chambers and passages were systematically searched, and bats were identified in situ using morphological characters [42]. Relative abundance per site was calculated as the proportion of each species relative to the total number of bats counted during that single visit.

**Figure 2:**
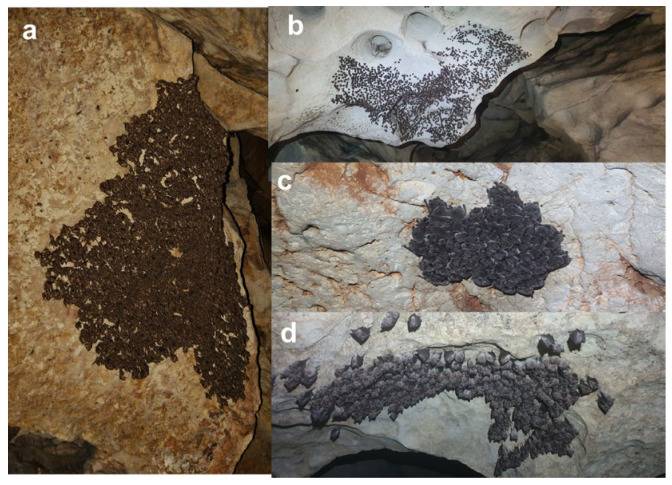
Cave-dwelling bat colonies in the studied karst caves in Bulgaria: (**a**) A large colony of *Miniopterus schreibersii* in Razhishka Cave; (**b**) one of the biggest colonies of *Rhinolophus euryale* in Magura Cave; (**c**) a cluster with *Myotis capaccinii* in Ivanova voda Cave; (**d**) a colony *of Rhinolophus ferrumequinum* in Andaka Cave.

For *Myotis myotis* and *M. blythii*, which are morphologically cryptic in hibernation, we recorded individuals as “*M. myotis/blythii* complex” unless diagnostic features were clearly visible. We minimized disturbance by using red LED lights and limiting observation time to <30 s per individual. Individuals were counted directly at the cave or, for larger bat clusters (Figure 2), using a photographic method [43]. The same counting methodology was used each year, or at each site per year, depending on site accessibility. We recorded species identity and count per species per site.

#### 2.2.2. Environmental Variables

We measured cave temperature and humidity using calibrated data loggers (Log 32 TH psychro-thermo-hygro- and temperature meter, TFA Dostmann GmbH & Co., Wertheim, Germany) placed in the main hibernation chamber of each cave, 1.5 m above the floor, away from entrances to avoid external influences. Loggers recorded temperature at 1-h intervals throughout the survey period (December 2020–March 2025). Therefore, we provide a cave-level thermal measurement rather than capturing fine-scale microsite variation.

We calculated the mean hibernation temperature for each cave by averaging all measurements during the core hibernation period (15 January–15 February) when bat counts were conducted. We focused on mean cave temperature as our primary environmental variable because temperature is the most widely documented driver of hibernation site selection [1,6], it varies predictably with elevation, and species-specific thermal preferences are well-established. We acknowledge that other factors (diurnal temperature variability, thermal amplitude, or vertical thermal gradients within caves, airflow velocity, chamber morphology) also influence hibernation site selection but were not systematically measured in this study due to logistical constraints of surveying 25 sites across a large geographic area. Cave length was initially considered a potential explanatory variable, but was excluded from the final analysis because bat colonies were consistently located near cave entrances and did not use the deeper sections of the caves.

#### 2.2.3. Bat Community Data

Bat assemblages were surveyed in 24 caves and 1 subterranean site in Bulgaria (Table 1). For each cave, species composition and abundance were recorded, resulting in a site × species matrix based on total bat abundance. Species richness was calculated per cave but was not included in multivariate community analyses.

#### 2.2.4. Multivariate Community Analysis

We constructed a site-by-species matrix with bat counts for each cave. We applied the Hellinger transformation to abundance data prior to ordination analyses to reduce the influence of rare species and double absences [44]. Elevation was treated both as a continuous variable and categorized into three zones (low, mid, high) based on natural breaks in the elevation distribution. We visualize the pattern of bat assemblage composition across the elevation gradient using non-metric multidimensional scaling (NMDS). To account for differences in total abundance among the subterranean sites studied, we used Bray–Curtis’s dissimilarity, the appropriate measure for community abundance data. To assess the relationship between community composition and environmental factors, we calculated Pearson correlation coefficients between the first two NMDS axes and the three environmental variables: temperature, humidity, and altitude. We computed the coefficient of determination (R^2^), the square of the correlation coefficient, to quantify the proportion of variance in the ordination axes explained by each environmental factor. We tested for differences in bat assemblage composition across elevation zones using permutational multivariate analysis of variance (PERMANOVA) based on Bray–Curtis dissimilarity matrices [45]. We used 9.999 permutations and assessed multivariate dispersion homogeneity using PERMDISP to ensure PERMANOVA assumptions were met [46]. Effect sizes were calculated as R^2^ values.

#### 2.2.5. Species-Level Analyses

Species-specific contributions to community differences were calculated by SIMPER analysis. We aimed to compare each elevation group, the average dissimilarity, the contribution of each species to that dissimilarity (as a percentage), and the cumulative percentage contribution. Only species that contribute to at least 80% of the cumulative dissimilarity for each comparison were reported, along with the mean abundances in each group. To combine a species’ relative abundance in a group (specificity) with its relative frequency of occurrence in that group (fidelity), we applied the IndVal index. Its values range from 0 (no indication) to 100 (perfect indication). Species with IndVal *p*-values ≤ 0.05 were considered significant indicators of their respective elevation groups.

For each cave, we determined three alpha diversity indices: Shannon diversity index (H’), Simpson diversity index (1-D), and species richness (the number of species). To evaluate differences in alpha diversity across elevation groups, we used a one-way ANOVA to compare mean diversity levels. The assumptions of normality and homogeneity of variance for ANOVA were assessed using Levene’s test. 

All statistical analyses were conducted using PAST (Paleontological Statistics) software version 5.3 [47]. Statistical significance was set at α = 0.05 for all tests.

## 3. Results

### 3.1. Bat Species Abundance and Elevation Distribution

We observed 9 bat species, totaling 70,712 individuals across 25 sites (Table 2). The number of bats per site ranged from 36 to 16.110, with an average of 7856.89 ± 6.635. The most abundant species was *R. euryale*, whereas the rarest was *M. emarginatus*. *R. blasii* was recorded at 389 m a.s.l., while *R. euryale* was most common between 130 and 389 m a.s.l., with some individuals found at 472 and 688 m a.s.l. *R. ferrumequinum* was present across almost all elevation zones, except at high altitudes between 1218 and 1336. *R. hipposideros* specimens were found at all surveyed elevations, except at very low (130 m a.s.l.) and very high elevations (1336 m a.s.l.). *R. mehelyi* preferred low elevations between 130 and 389 m a.s.l., while *M. capaccinii* and the *M. myotis/blythii* complex were the most abundant species at high elevation at 1336 m a.s.l. The rarest species, *M. emarginatus*, occupied elevations between 389 and 503 m a.s.l. *M. schreibersii* was recorded at elevations between 264 and 688 m a.s.l.

### 3.2. Bat Community Composition Pattern Across Elevation Gradients

**Figure 3:**
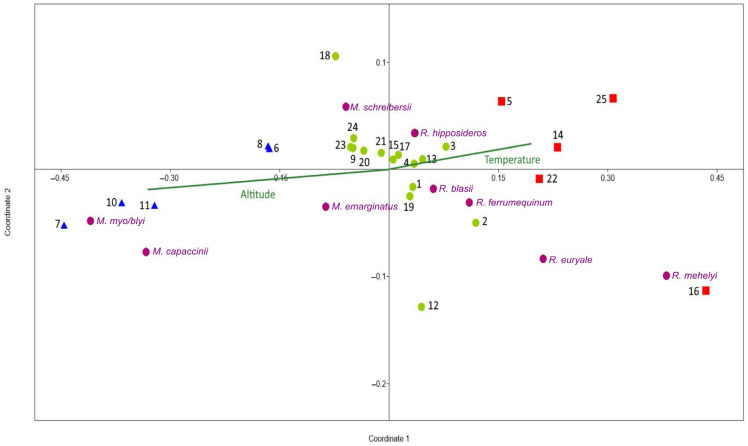
Non-metric multidimensional scaling (NMDS) ordination of cave-dwelling bat assemblages across three elevation categories. Red squares represent sampling sites at low elevation (130–300 m a.s.l.), green points at mid-elevation (323–511 m a.s.l.), and blue triangles at high elevation (≥680 m a.s.l.). The pink points represent the species. The ordination demonstrates a partial separation of assemblages along the elevational gradient, indicating gradual species replacement. The direction and strength of the correlations with the NMDS axes are indicated by environmental vectors. NMDS Coordinate 1 demonstrates a strong negative correlation with altitude (r = −0.994, *p* < 0.001) and a positive correlation with temperature (r = 0.805, *p* < 0.001).

Analysis of non-metric multidimensional scaling (NMDS) revealed a clear picture of the composition of bat communities across the three elevation categories. The NMDS ordination visualization demonstrated that the cave-dwelling bat assemblages studied showed partial separation among low-, mid-, and high-elevation sites. This suggests that species changes occur gradually along the elevation gradient (Figure 3). Correlating the NMDS coordinates with environmental variables showed that the main axis of variation in bat species (NMDS Coordinate 1) was significantly associated with elevation and related climatic conditions. Specifically, Coordinate 1 exhibited an extremely strong negative correlation with altitude (r = −0.994, *p* < 0.001, R^2^ = 0.988) and a strong positive correlation with temperature (r = 0.805, *p* < 0.001, R^2^ = 0.648). These relationships imply that the structure of bat assemblages is closely linked to altitude and that temperature changes similarly along this elevational axis. Together, these results imply that elevation and the associated temperature gradient are the dominant environmental factors influencing the composition of cave-dwelling bat communities. The strong negative link between Coordinate 1 and altitude, and the strong positive link with temperature, indicate an opposite relationship to that usually seen between elevation and temperature. There was a weak link between humidity and Coordinate 1, but it wasn’t significant (r = −0.201, *p* = 0.335, R^2^ = 0.04). NMDS Coordinate 2 showed no significant associations with any of the assessed environmental variables (temperature: r = −0.082, *p* = 0.698; humidity: r = −0.290, *p* = 0.160; altitude: r = 0.011, *p* = 0.958). This means that differences between bat colonies might be explained by factors we haven’t yet measured, such as the characteristics of the cave environment, the availability of roosting sites, or random events that led to colony formation.

### 3.3. Permutational Multivariate Analysis of Variance (PERMANOVA)

⁠⁠⁠⁠⁠⁠⁠PERMANOVA revealed significant differences in bat assemblage composition across elevation zones (F_2, 22_ = 1.616, *p* = 0.044, R^2^ = 0.128), indicating that 12.8% of compositional variance was explained by elevation grouping. Multivariate dispersion did not differ significantly among groups (PERMDISP: F_2, 22_ = 0.845, *p* = 0.442), confirming that PERMANOVA results reflect true location effects rather than differences in within-group variability. Pairwise PERMANOVA tests showed that bat species inhabiting caves at low elevation differed significantly from those at high and mid elevations (*p* = 0.046 and *p* = 0.0349, respectively), indicating species turnover along the elevation gradient. Nonetheless, no significant difference in species composition was observed between the mid- and high-elevation regions (*p* = 0.541), suggesting that these zones host species that are more similar. These results confirm that the elevation groups exhibited similar levels of within-group variability. This validates the interpretation of the PERMANOVA results as reflecting true differences in bat assemblages’ centroids rather than differences in dispersion.

### 3.4. Species Contributions to Elevation Group Dissimilarity

**Figure 4:**
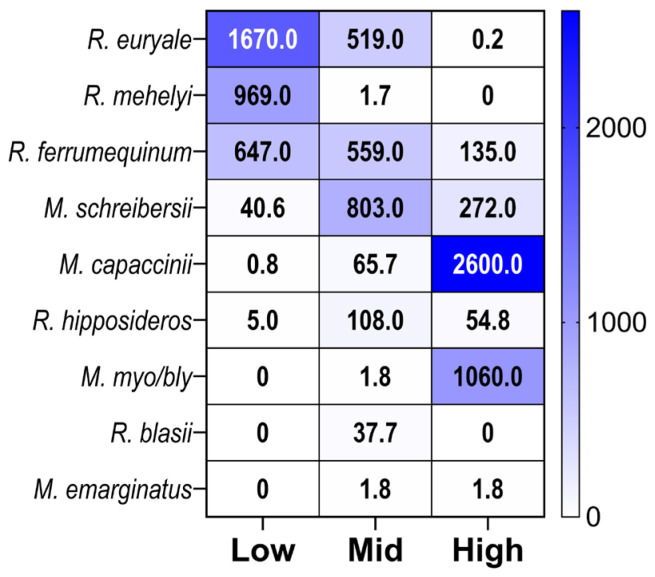
Species abundance heatmap showing the species clustering among elevation groups: low (130–300 m a.s.l.), mid-elevation (323–511 m a.s.l.), and high (≥680 m a.s.l.), matching temperature and humidity gradients. The color gradient and values in the boxes represent the average abundance.

A SIMPER analysis revealed that bat communities exhibited high overall dissimilarity (91.12%) across all pairwise comparisons, indicating significant variation in assemblage composition and species turnover along the elevation gradient. The analysis revealed several dominant species that largely explained variation among elevation groups, highlighting clear elevational zonation (Figure 4). Analysis of bat colonies at mid- and low elevations revealed that four species accounted for 94.17% of the total differences between the groups (Table 2). *Rhinolophus euryale* was the primary contributor to dissimilarity (40.66%), with substantially higher mean abundance at low elevations (mean = 1670 individuals) compared to mid elevations (mean = 519 individuals). This pattern identifies *R. euryale* as a characteristic species in bat assemblages at low elevations. *Rhinolophus ferrumequinum* contributed 32.03% to dissimilarity, showing moderately higher abundance at low elevations (mean = 647) than at mid elevations (mean = 559). In contrast, *Miniopterus schreibersii* contributed 13.02% to dissimilarity, with substantially higher abundance in mid-elevation assemblages (mean = 803) than in low-elevation assemblages (mean = 40.6), indicating that this species is characteristic of the studied mid-elevation colonies. *Rhinolophus mehelyi* contributed 8.46% to dissimilarity, with near-absence at mid elevations (mean = 1.73) but high abundance at low elevations (mean = 969), further characterizing the low-elevation assemblage.

The comparison between mid- and high-elevation sites revealed that four species accounted for 82.51% of the total dissimilarity (Table 3). *Miniopterus schreibersii* was the primary contributor at 32.1%, with much higher abundance at mid-elevations (average 803) than at high elevations (average 272), underscoring its specialization for mid-elevation habitats. *Rhinolophus ferrumequinum* contributed 24.04% to dissimilarity, showing higher abundance at mid elevations (mean = 559) than at high elevations (mean = 135). *Myotis capaccinii* contributed 15.21% to dissimilarity and showed a clear pattern of high-elevation specialization, with a mean abundance at high elevations (2600) that was more than 3 times that at mid elevations (65.7). It was the dominant species in high-elevation communities. *Rhinolophus hipposideros* contributed 11.16% to dissimilarity, with higher abundance at mid elevations (mean = 108) compared to high elevations (mean = 54.8).

The comparison of bat colonies at low and high elevations showed that four species accounted for 83. 98% of the overall dissimilarity, indicating notable shifts in species composition along the elevation gradient (Table 3). *Rhinolophus euryale* was the leading contributor (32.41%), with very high abundance at low elevations (mean = 1670) and almost absent at high elevations (mean = 0.2), reflecting a strong preference for low elevations. *Rhinolophus ferrumequinum* contributed 25.1%, with higher abundance at low elevations (mean = 647) compared to high elevations (mean = 135). *Myotis capaccinii* contributed 13.65%, displaying an opposite trend: nearly absent at low elevations (mean = 0.8) but highly abundant at high elevations (mean = 2600), highlighting it as a high-elevation specialist. *Miniopterus schreibersii* made up 12.81%, with low abundance at low elevations (mean = 40.6) and moderate abundance at high elevations (mean = 272).

### 3.5. Indicator Species Analysis

**Figure 5:**
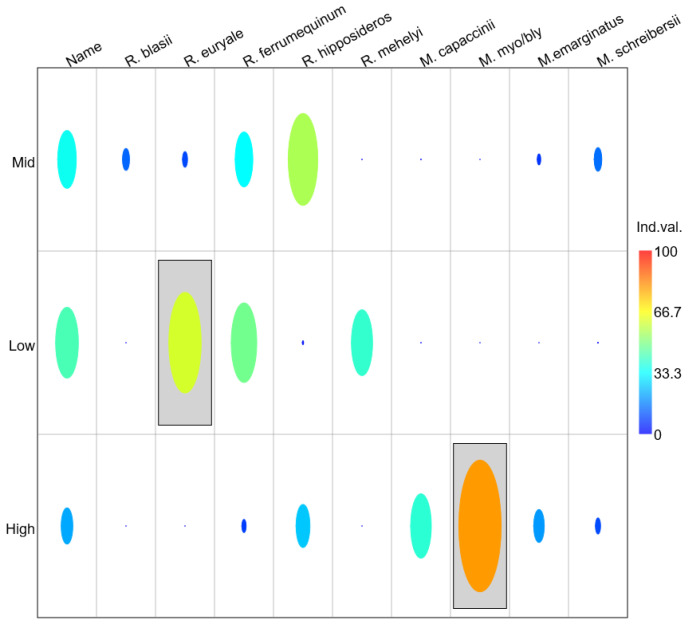
Heatmap of indicator species analysis (IndVal) of cave-dwelling bat assemblages across three elevation categories (Low, Mid, and High), performed in PAST version 5. Columns represent bat species and rows represent elevation groups: low (130–300 m a.s.l.), mid-elevation (323–511 m a.s.l.), and high (≥680 m a.s.l.). The size and color intensity of the ellipses indicate the indicator value (IndVal, %) for each species–elevation combination, with larger, warmer symbols representing stronger indicator relationships. Species highlighted by shaded boxes show the highest indicator values for a given elevation category.

Indicator Species Analysis (IndVal) revealed two species with significant fidelity to particular elevation groups, strongly supporting the idea of elevational specialization within the bat community (Figure 5). At low elevations, *Rhinolophus euryale* is a highly important indicator species (IndVal = 62.53%, *p* = 0.012), with both high abundance and frequent cave occurrence at these altitudes. This result confirms the SIMPER findings and establishes *R. euryale* as a reliable indicator of low-elevation bat communities. *Rhinolophus mehelyi* showed moderately high indicator value for low elevations (IndVal = 39.06%, *p* = 0.056), approaching but not reaching statistical significance. At mid elevations, no species showed significant indicator values. *Rhinolophus hipposideros* had the highest IndVal score for this group (39.37%, *p* = 0.237), followed by *Rhinolophus ferrumequinum* (26.49%, *p* = 0.552), but neither reached statistical significance. This suggests that mid-elevation communities are characterized by mixed assemblages rather than by species with strong fidelity to this elevation zone. At high elevations, *Myotis myotis/blythii* complex was identified as a highly significant indicator species (IndVal = 78.03%, *p* = 0.003), representing the strongest indicator value observed in the entire dataset. This result establishes the *M. myotis/blythii* complex as a reliable indicator of high-elevation bat communities. *Myotis capaccinii* showed moderately high indicator value for high elevations (IndVal = 35.94%, *p* = 0.085), approaching but not reaching statistical significance, though SIMPER analysis clearly identified this species as a dominant component of high-elevation assemblages. The remaining species showed low indicator values across all elevation groups, with non-significant *p*-values (all *p* > 0.10), indicating either broad elevational distributions or inconsistent occurrence patterns. The absence of significant indicator species for mid-elevation zones (all *p* > 0.05) suggests this zone represents a transition area where species from both low and high elevations overlap, consistent with ecotone dynamics.

### 3.6. Alpha Diversity Across Elevation Groups

Analysis of alpha diversity revealed no significant differences among elevation groups. Shannon diversity (H′) and Simpson diversity (1-D) did not vary significantly with elevation (one-way ANOVA: F_2, 22_ = 0.28, *p* = 0.76 and F_2, 22_ = 0.15, *p* = 0.86, respectively), and results were consistent across permutation-based and non-parametric tests. Assumptions of homogeneity of variance were met for both indices. These results indicate that overall alpha diversity of cave-dwelling bat assemblages remains relatively constant across the elevational gradient. Species richness varied widely among individual caves, ranging from 1 to 9 species per site, but no consistent pattern with elevation was evident. This pattern of constant alpha diversity coupled with high beta diversity suggests species replacement rather than species loss along the elevation gradient.

## 4. Discussion

### 4.1. Environmental Structuring of Bat Assemblages

Our results demonstrate that the primary environmental factor shaping hibernating bat assemblages in Bulgarian caves is the elevation-associated temperature gradient. This supports the hypothesis that thermal niche differentiation determines species distributions. Elevation-associated temperature gradients strongly structured the composition of the bat assemblages, with altitude and temperature explaining 32.4% of the variance in assemblage variation. Mean cave temperatures differed significantly across elevation zones (low: 10.55 °C; mid: 7.90 °C; high: 3.73 °C; *p* < 0.001), creating distinct thermal environments that filter species based on physiological tolerances. Assemblages at low and high elevations differed significantly. The mid-elevation assemblages differed from those at low elevation but not from those at high elevation and can be described as transitional. The observed pattern aligns with the environmental filtering hypothesis, suggesting that physiological temperature preferences limit species to elevations with suitable thermal conditions [48]. Heterothermic cave bats must manage the costs of thermoregulation while conserving energy during hibernation [49]. While humidity is often cited as important for bat distributions [50,51], we found no significant correlation between humidity and assemblage composition (r = −0.201, *p* = 0.335). This finding suggests that in the cave microhabitat, where high, constant relative humidity is common year-round, temperature variations exert stronger selective pressures than humidity gradients. This supports the notion that cave-dwelling bats generally adapt to high humidity, with temperature as the primary limiting factor in roost selection [4,52]. As a result, thermal niche differentiation is the primary factor shaping cave-dwelling bat communities along elevational gradients in Bulgaria.

### 4.2. Thermal Niche Differentiation Between Rhinolophus and Myotis Species

⁠⁠⁠SIMPER analysis showed that *Rhinolophus euryale* and *R. mehelyi* dominate the low-elevation group, with an average of 1670 and 969 individuals, respectively. Indicator Species Analysis confirmed *R. euryale* as a significant low-elevation indicator (IndVal = 62.53%, *p* = 0.0124). Rhinolophus species have relatively high metabolic rates and select warmer hibernacula (6–12 °C) that minimize the temperature differential between torpor and normothermia, reducing arousal costs [5,53]. Studies of *R. ferrumequinum* have found preferred roost temperatures of 8–12 °C during hibernation, with metabolic rates increasing substantially at lower temperatures [54]. The mean temperature at low elevations in our study (10.55 ± 1.38 °C) is in this optimal range, making conditions favorable for *Rhinolophus* species. In contrast, the mean temperature at high elevations (3.73 ± 1.59 °C) likely imposes costly thermoregulatory burdens on these warm-adapted species, which may explain their rarity in high-elevation caves.

On the other hand, *Myotis* species are adapted to cold environments by employing various physiological tactics, such as torpor, reduced metabolic rates, and thermal plasticity [13,55]. *M. myotis* hibernates at 2–4 °C, with periods of extended torpor at temperatures approaching 0 °C near-freezing [56,57]. This allows exploitation of colder sites, offering additional advantages, such as reduced pathogen activity and lower predation risk [58,59]. *Myotis capaccinii* and the *M. myotis/blythii* complex are prevalent in high-elevation cave assemblages, with average counts of 2600 and 1060 individuals, respectively. Based on Indicator Species Analysis, *M. myotis/blythii* complex is a highly significant indicator of high elevations (IndVal = 78.03%, *p* = 0.003). *Myotis* species distributions at high elevations reduce competition with *Rhinolophus* species through thermal niche separation. The distribution of *M. capaccinii* warrants particular attention. The species’ preference for high altitudes and cold caves contrasts with its Mediterranean range and its connection to aquatic foraging areas. This contradiction can be explained by the fact that Bulgaria lies within the northernmost range of the species, where cooler conditions during hibernation may provide optimal energy balance for a species adapted to warmer climates during the active season

Studies from temperate regions also show that thermal constraints on hibernation and torpor exert significant selective pressure on bat distributions [16,60]. Similarly, research on North American and European hibernating bat communities found that temperature was the key factor in roost selection and community composition [61,62]. However, tropical studies often prioritize precipitation, humidity and food availability over temperature, demonstrating that environmental drivers vary across latitudes [63,64]. The thermal niche differences observed between *Rhinolophus* and *Myotis* in our study reflect patterns seen in Europe, where *Rhinolophus* species are associated with warmer, lower-altitude sites and *Myotis* species with colder, higher-altitude roosts [65,66]. Similar elevational separation has been documented in the Alps, where *R. ferrumequinum* rarely inhabits areas above 1000 m, and *M. myotis* is rarely recorded above 2000 m [67]. Thus, it can be concluded that the distribution of cave-dwelling bats is driven by their physiological thermoregulatory processes. Similar patterns of thermal niche differentiation have been documented in tropical montane systems, where lowland species have been replaced by higher-elevation specialists [68]. This aligns with the metabolic theory of ecology, which holds that species’ distributions are primarily determined by their basic physiological needs rather than by interactions with other species [69].

### 4.3. Species Turnover Rather than Diversity Gradients

No significant difference in diversity was observed among elevation groups, despite notable species turnover. Shannon diversity (ANOVA F = 0.279, *p* = 0.759) and Simpson diversity (ANOVA F = 0.146, *p* = 0.865) indexes showed no significant differences among low, mid, and high elevations. This contrasts with high overall dissimilarity (SIMPER = 91.12%) and significant PERMANOVA (*p* = 0.044), indicating that elevational turnover involves species replacement rather than changes in richness or evenness. Several studies have also reported this notable change in species composition with elevation [50,70,71]. This challenges the classic view that diversity declines monotonically with elevation, a pattern documented across groups such as birds, small mammals, and insects [72,73,74]. The species-replacement pattern without diversity change indicates that bat assemblages are mainly structured by species-specific temperature tolerances rather than by richness-driven processes [75]. Each elevation hosts a unique species adapted to its temperature, with a stable number of co-occurring species that maintains species richness across zones [76].

Several hypotheses of mid-domain effect, area effects and productivity [77,78] have been proposed to explain elevational diversity gradients. However, our results indicate that none of these mechanisms produce strong diversity gradients in cave-dwelling bats across elevations of 100–1334 m, which differs from the conclusions of some previous studies. The high turnover observed in our study, with an overall dissimilarity of 91.12%, was driven only by four thermally specialized species and exceeded that reported in many tropical research efforts. This likely reflects the more extreme temperature differences typical of temperate cave environments, rather than tropical forests, where vegetation buffers temperature fluctuations [68]. In the Andes and the mountains of Southeast Asia, the greatest species diversity was found at mid-elevations, which appear to be transition zones where the distributions of species from lowlands and highlands overlap [73,79]. The specific, limiting microhabitats found in caves may reduce the number of species that can coexist at each elevation, potentially lowering the diversity typically observed in structurally more complex forest habitats [80].

Consequently, thermal gradients become the dominant factor of niche differentiation, with each elevation zone supporting a relatively fixed number of thermally adapted species. This pattern highlights beta diversity as the primary component of regional diversity in cave-dwelling bats, with important implications for prioritizing habitat connectivity across elevations rather than just hotspots.

### 4.4. Mid-Elevation as an Ecotone and Transition Zone

Mid-elevation assemblages functioned as ecotones, differing significantly from low-elevation communities (*p* = 0.035) but not from high-elevation assemblages (*p* = 0.54). The absence of significant indicator species for mid-elevations (highest IndVal = 39. 37% for *R. hipposideros*, *p* = 0.242) contrasts sharply with the strong indicators for low (*R. euryale*) and high (*M. myotis/blythii* complex) elevations, suggesting that mid-elevation caves support mixed assemblages rather than distinct, specialized ones. The high spatial and temporal variability in the ecotones often supports species from adjacent assemblages as well as ecotone specialists [81,82]. The absence of significant mid-elevation indicators in our study suggests that this zone lacks true ecotone specialists and instead represents an overlap zone where species from both low and high elevations co-occur at reduced abundances. *Miniopterus schreibersii* was the dominant species in the mild-elevation caves (mean = 803 individuals). Nevertheless, this species showed intermediate abundance, higher than in the low-elevation caves (mean = 40.6) but lower than in some high-elevation specialists. The broad temperature tolerance of *M. schreibersii* [83,84] and its significant variation in roosting behavior enable it to utilize transitional temperature zones effectively, to which it is well adapted. The mean temperature at mid-elevations in our study (7.90 ± 1.62 °C) is suboptimal for both warm-loving *Rhinolophus* and cold-adapted *Myotis* species, but suitable for temperature-flexible generalists such as *M. schreibersii*.

The characterization of mid-elevation zones as overlap zones is consistent with the relatively high standard deviation in temperature at mid elevations (±1.62 °C), which exceeds that at low elevations (±1.38 °C) and approaches that at high elevations (±1.59 °C), suggesting greater thermal heterogeneity in this transitional zone. Mid-elevation ecotones are of high ecological importance because they serve not only as transition zones but also as temperature refugia during climatic extremes. They provide other roosting sites when the weather is too hot or cold at high altitudes [85]. Furthermore, caves at mid-elevations might enable gene flow between populations at lower and higher elevations, helping to preserve genetic connectivity along the elevation gradient [86]. The absence of a notable difference between mid- and high-elevation communities (*p* = 0.541) indicates that mid-elevation caves might be more closely linked to high-elevation assemblages. Although [87] forecast latitudinal expansion, our current findings suggest shifts in altitude. While the geographic range could increase, the quality of hibernation sites, particularly stable cold caves, may decline, leading to a complex trade-off between range and habitat suitability. This connection could allow mid-elevation caves to serve as corridors for cold-adapted species expanding their ranges downhill during cooler periods. The positioning of this ecotone in the mid-elevation zone may reflect the balance between species’ upper and lower thermal limits, with warm-adapted species reaching their cold tolerance limits while cold-adapted species can still persist in moderately warm conditions.

### 4.5. Physiological Limitations

The distribution of cave-dwelling bats along elevation gradients described in this study likely illustrates that it depends on thermoregulation, energy balance, and hibernation. Bats are heterothermic endotherms capable of facultative torpor, which helps conserve energy during times of food scarcity or thermal stress [49,88]. However, the ability to enter torpor varies widely among species, with significant implications for elevation-related adaptations and distributions. *Rhinolophus* species have a more limited ability to enter torpor than *Myotis* species, maintaining higher minimum body temperatures during torpor and waking more often [5]. Research on *R. ferrumequinum* shows that their lowest body torpor temperatures range from 5–8 °C, with individuals waking spontaneously if ambient temperatures dip below this range [54]. These physiological limitations lead to higher energetic costs at cold, high-altitude locations, where frequent arousals drain fat reserves and decrease overwinter survival [89]. In our study, the average temperature at high elevations (3.73 ± 1.59 °C) is below the minimum torpor temperature for most *Rhinolophus* species, which accounts for their almost complete absence from these sites. In contrast, *Myotis* species exhibit greater cold tolerance, with some able to sustain torpor at body temperatures near 0 °C [55]. *M. lucifugus*, a North American relative of *M. myotis*, has been observed hibernating at ambient temperatures as low as −5 °C, with body temperatures dropping to 2 °C [90,91]. This remarkable ability allows *Myotis* species to inhabit high-elevation caves that stay cold throughout hibernation, thereby lowering thermoregulatory energy costs and prolonging torpor. The prevalence of *M. capaccinii* and *M. myotis/blythii* at high altitudes illustrates these physiological adaptations.

While our data demonstrate that temperature is the primary driver of elevational distributions, other factors, including food availability [92], predation risk [93], and disease transmission [94], may interact with thermal constraints. However, the high abundances we observed at high elevations (e.g., *M. capaccinii*) suggest that food limitation is not the primary constraint during hibernation, when bats rely on stored fat reserves rather than active foraging.

### 4.6. Climate Change Implications

Our findings have important implications for predicting bat responses to climate change. Understanding these responses requires considering both the energetic basis of thermal niche selection and the buffering capacity of cave microclimates. Warm-preferred species (*R. euryale*, *R. mehelyi*) may expand into currently mid- and high-elevation caves as temperatures increase, while cold-adapted species (*M. capaccinii*, *M. myotis/blythii*) may face range contractions as suitable cold sites become scarce. The strong restriction of high-elevation species to cold caves (mean 3.73 °C) suggests potential thermal specialization. If high-elevation species have developed narrower thermal tolerance ranges than low-elevation generalists, a common pattern among mountain taxa [95], they are at greater risk of extinction as their suitable habitats shrink.

High-elevation caves currently provide critical cold refugia. As the coldest sites warm, species requiring temperatures < 4 °C may lose all suitable hibernation habitat, with no opportunity to move upslope beyond the highest peaks. The mid-elevation ecotone may shift upward, compressing the range of high-elevation specialists. The rate of this shift will depend on cave-specific thermal buffering capacity, which varies with cave morphology and depth. However, the magnitude and rate of these changes will depend critically on cave-specific microclimate buffering capacity. Due to their thermal mass and heat exchange with the underlying rock, caves exhibit thermal capacity, which can mitigate external temperature fluctuations [96,97]. Deep caves with restricted entrances may buffer surface warming more effectively than shallow or well-ventilated caves, potentially serving as climate refugia [97]. The energetic consequences of warming are species-specific. Even small temperature increases (1–2 °C) can substantially increase metabolic costs during torpor, reducing fat reserves and overwinter survival [14,98]. Species that hibernate near their upper thermal tolerance limits may experience increased arousal frequency and energy depletion.

Earlier spring warming may trigger premature arousal from hibernation before insect prey are available, increasing the risk of starvation [99]. Species with narrow thermal niches and limited dispersal ability (e.g., *M. capaccinii* in Bulgaria) face elevated extinction risk as suitable habitat becomes fragmented or disappears entirely. Long-term monitoring of cave temperatures and bat assemblages, combined with thermal physiology and energetics, is needed to predict which caves will serve as climate refugia and which species are most vulnerable to warming [22,26].

### 4.7. Limitation of the Study

Several limitations warrant consideration. First, our elevation gradient is limited to 1334 m, and studying higher elevations may reveal additional patterns. Second, our single-visit survey design provides a snapshot of hibernation assemblage composition but may miss temporal variation in cave use throughout the winter. Some species may move between caves during hibernation, and our counts represent presence at one time point rather than total seasonal use. The strong association between temperature and species composition suggests it is a primary structuring factor, but not the only one. Other factors, such as airflow velocity, thermal stability (temperature variance), and chamber topography, also influence bats’ hibernation site selection, and may explain some of the unexplained variance in our models (67.6% of compositional variance remains unaccounted for by temperature and elevation). We also interpret our results as reflecting thermal microclimate selection during hibernation rather than landscape-level filtering, because bats actively select hibernation sites based on temperature when landscape factors are irrelevant (winter, no foraging), and species-specific thermal preferences are well-documented [14,58]. However, we acknowledge that elevation correlates with multiple environmental gradients, and landscape-level factors may also influence regional species distributions. Our cave-level study design demonstrates that temperature strongly predicts assemblage composition during hibernation, but it cannot separate landscape filtering from microhabitat selection at broader spatial scales.

## 5. Conclusions

Our results demonstrate that the primary environmental factor shaping hibernating bat assemblages in Bulgarian caves is the elevation-associated temperature gradient. The unique thermal niches differentiation drives significant species turnover (91.12% dissimilarity). The strong differentiation between warm-adapted *Rhinolophus* and cold-adapted *Myotis* species, combined with elevation-specific indicator species and the absence of mid-elevation specialists, reveals that thermal tolerance, rather than interspecific competition or diversity gradients, determines assemblage composition. This pattern emphasizes beta diversity as the main element of regional diversity among cave-dwelling bats.

These findings are crucial for understanding and addressing climate change predictions. As warming progresses, cold-adapted high-elevation specialists (*M. capaccinii*, *M. myotis/blythii* complex) face range contractions and potential extirpation, while warm-adapted species (*R. euryale*, *R. mehelyi*) may expand upward. Mid-elevation ecotones will likely shift upward, compressing suitable habitat for cold-adapted species and potentially eliminating thermal refugia at the highest elevations. Landscape-scale approaches that preserve habitat connectivity across the entire elevation range should be the focus of future conservation strategies. This will allow cave networks across various thermal environments stay protected and accessible.

Future research should address key gaps such as temporal replication across several winters to evaluate seasonal and yearly cave use and assemblage stability; expanding elevational sampling beyond 1334 m to find high-elevation specialists and upper thermal limits; combining cave morphology, depth, and microclimate to improve thermal buffering predictions under warming; and genetic analyses to examine population connectivity and gene flow across elevation gradients. These efforts will improve our capacity to predict range shifts, pinpoint vulnerable populations, and guide targeted conservation efforts for Europe’s cave-dwelling bats amid rapid environmental changes.

## Figures and Tables

**Table 2:** Cave-specific absolute abundance and total counts of nine bat species in the Stara Planina and Rhodope Mountains.

Cave Name	Cave ID	*R. blasii*	*R. euryale*	*R. ferrumequinum*	*R. hipposideros*	*R. mehelyi*	*M. capaccinii*	*M. myo/bly*	*M. emarginatus*	*M. schreibersii*
Aina-Ini Cave	1	539	78	0	0	0	0	0	0	0
Andaka Cave	2	0	300	4136	1	0	0	0	0	896
Bacho Kiro Cave	3	0	0	105	271	0	0	0	0	0
Cave next to Samara Cave	4	0	1	0	2	0	0	0	0	0
Emenska cave	5	0	9	199	2	1	4	0	0	203
Golak mine	6	0	1	3	9	0	0	0	0	170
Ivanova Voda Cave	7	0	0	0	0	0	15,000	5267	0	0
Kalotinska Temna Dupka Cave	8	0	0	36	128	0	0	1	0	1190
Kazarskata Cave	9	0	0	0	27	0	0	0	0	0
Lednikat Cave	10	0	0	3	1	0	0	1	2	0
Lepenitsa Cave	11	0	0	633	136	0	3	16	7	0
Magura Cave	12	0	7373	2716	1126	0	959	0	1	0
Ringova Pesht Cave	13	0	0	1	7	0	0	0	0	0
Mandrata Cave	14	0	1000	432	18	0	0	0	0	0
Mikrenska Cave	15	0	0	300	0	0	0	0	0	0
Orlova Chuka Cave	16	0	6321	144	0	4842	0	0	0	0
Ovcharskata Cave	17	0	0	4	1	0	0	1	0	0
Razhishka Cave	18	0	0	1059	18	0	0	0	0	11,125
Samara Cave	19	26	26	26	26	26	26	26	26	26
Svinskata dupka cave	20	0	1	4	115	0	0	0	0	0
Temnata Dupka Cave	21	0	0	14	11	0	0	0	0	0
Urushka-Maara Cave	22	0	0	2459	4	0	0	0	0	0
Vrazhi dupki 14 (Prilepska) cave	23	0	0	18	11	0	0	0	0	0
Vrazhi dupki 9 cave	24	0	0	7	1	0	0	0	0	0
Zandana Cave	25	0	1000	3	1	0	0	0	0	0
Total		565	16,110	12,302	1916	4869	15,992	5312	36	13,610

**Table 3:** Top contributing species by elevation groups comparison.

Comparison of Elevation Groups	Rank	Species	Contribution %	Cumulative %	Abundance Pattern
Mid vs. Low	1	*R. euryale*	40.66	40.66	Low >> Mid (1670 vs. 519)
	2	*R. ferrumequinum*	32.03	72.69	Low > Mid (647 vs. 559)
	3	*M. schreibersii*	13.02	85.71	Mid >> Low (803 vs. 40.6)
	4	*R. mehelyi*	8.46	94.17	Low >> Mid (969 vs. 1.73)
Mid vs. High	1	*M. schreibersii*	24.04	56.14	Mid >> High (559 vs. 135)
	2	*R. ferrumequinum*	15.21	71.35	High >> Mid (2600 vs. 65.7)
	3	*M. capaccinii*	11.16	82.51	Mid > High (108 vs. 54.8)
	4	*R. hipposideros*	24.04	56.14	Mid >> High (559 vs. 135)
Low vs. High	1	*R. euryale*	32.41	32.41	Low >> High (1670 vs. 0.2)
	2	*R. ferrumequinum*	25.10%	57.51	Low >> High (647 vs. 135)
	3	*M. capaccinii*	13.65	71.16	High >> Low (2600 vs. 0.8)
	4	*M. schreibersii*	12.81	83.98	High > Low (272 vs. 40.6)

Note: “>>“ indicates major difference (>5-fold); “>“ indicates moderate difference (2–5-fold).

## Data Availability

The data that support the findings of this study are available from the corresponding authors upon reasonable request.
